# The burden of splice-disrupting variants in inherited heart disease and unexplained sudden cardiac death

**DOI:** 10.1038/s41525-023-00373-w

**Published:** 2023-10-11

**Authors:** Emma S. Singer, Joshua Crowe, Mira Holliday, Julia C. Isbister, Sean Lal, Natalie Nowak, Laura Yeates, Charlotte Burns, Sulekha Rajagopalan, Ivan Macciocca, Ingrid King, Julie Wacker, Jodie Ingles, Robert G. Weintraub, Christopher Semsarian, Richard D. Bagnall

**Affiliations:** 1 Agnes Ginges Centre for Molecular Cardiology at Centenary Institute, The University of Sydney, Sydney, NSW Australia; 2 Faculty of Medicine and Health, The University of Sydney, Sydney, NSW Australia; 3 Department of Cardiology, Royal Prince Alfred Hospital, Sydney, NSW Australia; 4 Cardio Genomics Program at Centenary Institute, The University of Sydney, Sydney, NSW Australia; 5 Centre for Population Genomics, Garvan Institute of Medical Research, and UNSW, Sydney, NSW Australia; 6 Centre for Population Genomics, Murdoch Children’s Research Institute, Melbourne, VIC Australia; 7 Department of Clinical Genetics, Liverpool Hospital, Sydney, NSW Australia; 8 Murdoch Children’s Research Institute, University of Melbourne, Melbourne, VIC Australia; 9 Victorian Clinical Genetics Services, Melbourne, VIC Australia; 10 University of Melbourne, Melbourne, VIC Australia; 11 Department of Cardiology, Royal Children’s Hospital, Melbourne, VIC Australia

**Keywords:** Genetic testing, Medical genetics

## Abstract

There is an incomplete understanding of the burden of splice-disrupting variants in definitively associated inherited heart disease genes and whether these genes can amplify from blood RNA to support functional confirmation of splicing outcomes. We performed burden testing of rare splice-disrupting variants in people with inherited heart disease and sudden unexplained death compared to 125,748 population controls. ClinGen definitively disease-associated inherited heart disease genes were amplified using RNA extracted from fresh blood, derived cardiomyocytes, and myectomy tissue. Variants were functionally assessed and classified for pathogenicity. We found 88 in silico-predicted splice-disrupting variants in 128 out of 1242 (10.3%) unrelated participants. There was an excess burden of splice-disrupting variants in *PKP2* (5.9%), *FLNC* (2.7%), *TTN* (2.8%), *MYBPC3* (8.2%) and *MYH7* (1.3%), in distinct cardiomyopathy subtypes, and *KCNQ1* (3.6%) in long QT syndrome. Blood RNA supported the amplification of 21 out of 31 definitive disease-associated inherited heart disease genes. Our functional studies confirmed altered splicing in six variants. Eleven variants of uncertain significance were reclassified as likely pathogenic based on functional studies and six were used for cascade genetic testing in 12 family members. Our study highlights that splice-disrupting variants are a significant cause of inherited heart disease, and that analysis of blood RNA confirms splicing outcomes and supports variant pathogenicity classification.

## Introduction

Inherited cardiomyopathies and arrhythmia syndromes are important causes of heart failure and sudden cardiac death^1,2^. People with suspected inherited heart diseases should undergo genetic testing as this can clarify uncertain clinical diagnoses, guide treatment options, inform prognosis, and help stratify risk in family members^3^. While genetic testing typically focuses on rare variants in protein-coding regions and the essential splice sites of genes, there is an increasing appreciation of the range and manner by which variants can disrupt RNA splicing^4–6^. This includes variants previously annotated as missense, nonsense, or synonymous changes and intronic variants located far away from protein-coding regions. Assigning the correct consequence of variants at the protein level is essential for the accurate classification of pathogenicity and for understanding disease mechanisms.

RNA splicing requires the recognition of the splice donor and acceptor site at the beginning and end of introns (Supplementary Fig. 1a). The donor site is defined as the last three nucleotides of an exon and the first six nucleotides of the intron, with an almost invariant ‘GT’ dinucleotide at the beginning of the intron. The acceptor site comprises a polypyrimidine tract around the last 20 nucleotides of the intron and includes the first three nucleotides of the exon with an almost invariant ‘AG’ dinucleotide at the end of the intron^7,8^. Whilst in silico tools can predict whether a variant alters splicing with modest positive predicted values^9^, the outcomes can only be confirmed with functional studies involving amplification and sequencing of the mRNA (Supplementary Fig. 1b).

Rare variants at the essential splice dinucleotides of genes where loss-of-function is an established disease mechanism are usually assigned a pathogenic very strong (PVS1) criterion by the American College of Medical Genetics and Association for Molecular Pathology (ACMG/AMP) variant classification guidelines^10^. Consequently, these variants often classify as likely pathogenic or pathogenic. In contrast, most variants in the extended splice site regions, or those predicted to create new splice sites, classify as variants of uncertain significance (VUS) due to the uncertainty about if and how they disrupt splicing. Previous work has shown that functional studies confirming aberrant splicing can provide supportive evidence of pathogenicity and can aid the classification of splicing variants as likely pathogenic^5,11^. These functional studies have begun to reveal the types of splice-disrupting variants in selected genes; however, there is an incomplete knowledge of the range and burden of putative splice-disrupting variants in definitively disease-associated inherited heart disease genes across the different diseases, and it is unclear if these genes can be amplified from blood RNA to facilitate functional studies.

Here, we sought to describe the burden of in silico predicted, splice-disrupting variants in definitively disease-associated genes across a large clinical cohort of people with inherited heart disease or unexplained sudden cardiac death (SUD) compared to population controls. We also show which of the 32 definitively disease-associated Clinical Genome Resource (ClinGen) curated cardiac genes can be amplified from blood RNA to support functional studies, and we confirm the outcomes of splicing variants in the participant’s blood RNA, where available.

## Results

**Fig. 1:**
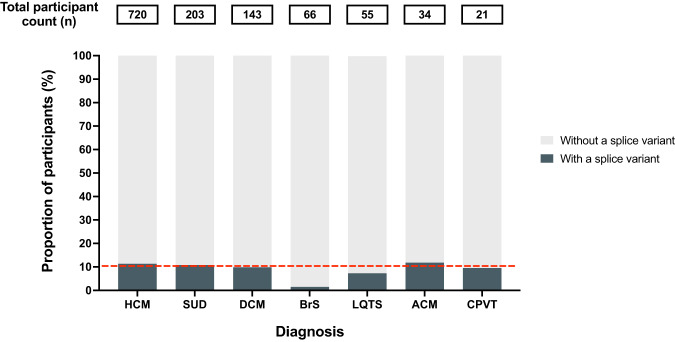
The proportion of participants with an in silico-predicted splice-disrupting variant. Total participant counts within each disease group are shown in the boxes above each bar plot. The overall average of participants with an in silico-predicted splice-disrupting variant (10.3%) is highlighted by the red dotted line. HCM hypertrophic cardiomyopathy, SUD sudden unexplained death, DCM dilated cardiomyopathy, BrS Brugada syndrome, LQTS long QT syndrome, ACM arrhythmogenic cardiomyopathy, and CPVT catecholaminergic polymorphic ventricular tachycardia.

**Fig. 2:**
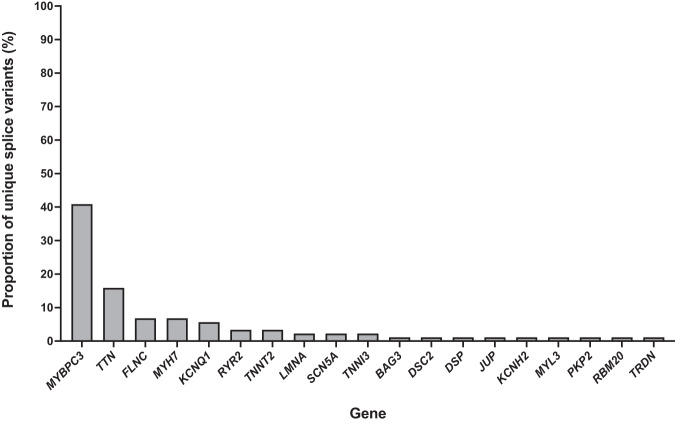
The percentage of unique in silico-predicted splice-disrupting variants in each gene. Percentage of unique in silico-predicted splice-disrupting variants found in a list of 32 genes prioritised based on phenotypically concordant genes established by the ClinGen Curation Expert groups for hypertrophic cardiomyopathy, dilated cardiomyopathy, arrhythmogenic cardiomyopathy, long QT syndrome, Brugada syndrome and catecholaminergic polymorphic ventricular tachycardia.

The burden of in silico predicted, splice-disrupting variants in definitively associated and phenotypically concordant disease genes was evaluated in 1242 unrelated participants with inherited heart disease or SUD. Hypertrophic cardiomyopathy (HCM) was the most common diagnosis in the cohort (*n* = 720), followed by SUD (*n* = 203), dilated cardiomyopathy (DCM) (*n* = 143), Brugada syndrome (BrS) (*n* = 66), long QT syndrome (LQTS) (*n* = 55), arrhythmogenic cardiomyopathy (ACM) (*n* = 34) and catecholaminergic polymorphic ventricular tachycardia (CPVT) (*n* = 21). We found 88 rare in silico predicted, splice-disrupting variants in 128 out of 1242 (10.3%) participants, and only nine participants had an alternative genetic cause for their disease (Supplementary Table 1). Within each disease group studied, approximately 10% of participants carried an in silico-predicted splice-disrupting variant, except for BrS, where only 1 out of 66 participants (1.5%) did (Fig. 1). The genes with the most splice-disrupting variants were *MYBPC3* (36/88, 41%), followed by *TTN* (14/88, 16%), *FLNC* (6/88, 7%), *MYH7* (6/88, 7%) and *KCNQ1* (5/88, 6%), with few variants, or none, in the remaining genes (Fig. 2). Self-reported ethnicities were available for 122 participants with splice-disrupting variants, with 95 European, 12 Asian, 6 North African, 8 Oceanian, and 1 ‘Peoples of the Americas’ (Supplementary Table 1).

We compared the burden of rare putative splice-disrupting variants in our disease cohorts with gnomAD (v2.1.1) exomes control populations. There was a significant excess of splice-disrupting variants in *PKP2* in people with ACM (excess burden in cases = 5.9%, *P* < 0.001), *FLNC* (2.7%, *P* < 0.001) and *TTN* (2.8%, *P* < 0.001) in people with DCM, *MYBPC3* (8.2%, *P* < 0.001) and *MYH7* (1.3%, *P* < 0.001) in people with HCM, and *KCNQ1* in people with LQTS (3.6%, *P* < 0.001) (Supplementary Table 2). Statistical significance of these six genes was maintained when repeating the burden test with only European cases and European Non-Finnish gnomAD controls (Supplementary Table 3). The excess of putative splice-disrupting *MYH7* variants is primarily driven by six HCM participants having a Glu849Gly missense change due to a c.2681 A > G variant at the second nucleotide of exon 23, which was predicted to disrupt splicing by the in silico tools, adapting boosting (ADA) and random forests (RF)^12^.

### Mapping the location of splice-disrupting variants

**Fig. 3:**
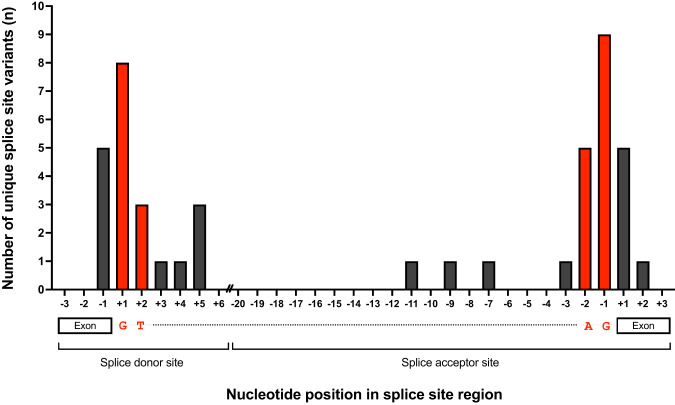
Location of unique in silico-predicted splice-disrupting variants in the essential splice site region. The positions of the essential GT/AG dinucleotides are shown in red. The splice donor site consists of the last three nucleotides of the exon and the first six nucleotides of the intron. The acceptor site consists of the last 20 nucleotides of the intron and the first three nucleotides of the exon. The intervening intron is truncated ‘//’. Three small deletions within the splice site region are not shown.

The location of in silico predicted, splice-disrupting variants within the donor and acceptor sites was assessed (Fig. 3, Supplementary Table 4). Twenty-four variants (27%) were in the donor site, of which 13 variants disrupted the essential ‘GT’ dinucleotide and three clustered at the +5 intronic nucleotide position. Twenty-four variants (27%) were in the acceptor site, of which 14 disrupted the essential ‘AG’ dinucleotide. Ten variants (11%) in the first and last nucleotide of an exon previously annotated as missense changes were predicted to disrupt the adjacent splice site.

**Fig. 4:**
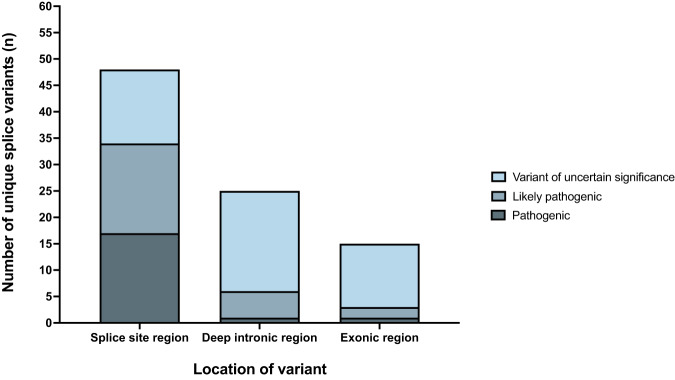
Classification of in silico-predicted splice-disrupting variants. Variants located in the −3 to +6 region of the donor site or the −20 to +3 region of the acceptor site are classified as ‘Splice site region’ variants. All other intronic variants were labelled as ‘deep intronic region’. Remaining variants in the exons, including missense, nonsense, and synonymous variants, were categorised as ‘exonic region’.

Outside of the canonical splice sites, there were 25 variants in deep intronic regions predicted to create new donor (*n* = 15) or acceptor (*n* = 10) sites, and 15 exonic variants annotated as missense, nonsense, or synonymous variant, predicted to create new donor (*n* = 11) or acceptor (*n* = 4) sites (Fig. 4, Supplementary Table 4).

### RNA sources for amplification of definitively disease-associated cardiac genes

We determined which sources of mRNA would support RT-PCR amplification of the definitively associated inherited heart disease genes. In total, 21 out of 31 (68%) genes were amplified concordantly using mRNA extracted from blood, induced pluripotent stem cell-derived cardiomyocytes and myectomy tissue (Supplementary Table 5, Supplementary Fig. 2). Nine genes only amplified in induced pluripotent stem cell-derived cardiomyocytes and myectomy tissue, (*CASQ2*, *TECRL*, *FLNC*, *MYH7*, *TNNT2*, *ACTC1*, *MYL2*, *MYL3* and *TNNI3*), while *TRDN* only amplified in myectomy tissue. Seven genes amplified more than one product due to alternative splicing (*RYR2, TECRL, BAG3, MYH7, ACTC1, TNNI3 and TPM1)* (Supplementary Fig. 2). *PLN*, which is definitively associated with DCM, was not included as the single coding exon in the MANE transcript, NM_002667.5, does not undergo splicing.

### Functional studies of in silico-predicted splice-disrupting variants

Six in silico predicted, splice-disrupting variants without prior mRNA testing were functionally studied using blood RNA from the affected individuals and family members who carried the variant, where available (Table 1). We assessed three variants in donor splice sites. Amplification of mRNA from a female with LQTS and a *KCNQ1* c.477+5 G > A variant revealed exon 2 skipping resulting in a 91 bp deletion, leading to a frameshift and premature termination codon (Supplementary Fig. 3a). An *RYR2* c.848+1 G > A variant in a female diagnosed with familial CPVT caused exon 11 skipping, leading to an in-frame deletion of 25 amino acids (Supplementary Fig. 3b). A *TTN* c. 63793 G > A missense variant at the last nucleotide of exon 307 was found in a male with SUD, his uncle with DCM, and his clinically unaffected father, totalling six meiosis. RNA extracted from the father showed the retention of intron 307, leading to a frameshift and a premature stop codon (Supplementary Fig. 3c). We assessed two variants in acceptor splice sites. A missense variant in *KCNQ1* c.781 G > A, found in a female with LQTS, caused the skipping of exon 6, leading to an in-frame deletion of 47 amino acids (Supplementary Fig. 3d). An *MYBPC3* c.1458-7 C > A variant found in a male with HCM caused a 5 bp extension of exon 17, leading to a premature stop (Supplementary Fig. 3e). Finally, a deep intronic c.1224-80 G > A variant in *MYBPC3* found in a male with HCM created a new splice acceptor site resulting in a 78 bp extension of exon 14, leading to an in-frame inclusion of 26 amino acids (Supplementary Fig. 3f).

**Table 1:** Functional study outcomes and sequence variant classification.

Family (diagnosis)	Variant	MES/ADA RF/SpliceAI	ClinVar ID (class)	Functional study outcome	ACMG classification without RNA analysis	ACMG classification with RNA analysis	Change in classification
014 (LQTS)	*KCNQ1* c.477+5 G > A	−5.37/0.99 0.97/0.23	53047 (P)	Exon 2 skipped (PTC)	VUS (PS4_moderate, PM2, PP1, PP3)	LP (PS3, PS4_moderate, PM2, PP1)	VUS → LP
015 (LQTS)	*KCNQ1* c.781 G > A	1.81/0.91 0.61/0.76	53103 (VUS)	Exon 6 skipped (in-frame deletion)	VUS (PM2, PS4_supporting, PP3)	VUS (PM2, PM4, PS4_supporting)	No change (VUS)
051 (HCM)	*MYBPC3* c.1224-80 G > A	7.96/NA NA/0.94	693982 (-)	Exon 14 extension (in-frame insertion)	VUS (PS4, PP3)	LP (PS4, PM2, PM4)	VUS → LP
054 (HCM)	*MYBPC3* c.1458-7 C > A	−3.93/0.99 0.45/0.99	–	Exon 17 extension (PTC)	VUS (PM2, PP3)	LP (PS3, PM2)	VUS → LP
102 (CPVT)	*RYR2* c.848+1 G > A	−8.18/1.0 0.94/0.74	201371 (Conflicting)	Exon 11 skipped (in-frame deletion)	LP (PP1_strong, PM2, PS4_supporting)	LP (PP1_strong, PM2, PM4, PS4_supporting)	No change (LP)
125 (SUD)	*TTN* c.63793 G > A	−4.57/1.0 0.99/0.74	202779 (VUS)	Intron retention (PTC)	LP (PP1_moderate, PM2, PS4_supporting, PP3)	LP (PS3, PP1_moderate, PM2, PS4_supporting)	No change (LP)

ACMG/AMP critieria: PS4 - ≥ 15 probands of concordant phenotype; PS4_moderate - ≥ 6 probands of concordant phenotype; PS4_supporting - ≥ 2 probands of concordant phenotype; PS3 - functional evidence with out-of-frame change; PM2 - rarity in the population; PM4 - functional evidence with in-frame change; PP1_strong - ≥ 7 meiosis; PP1_moderate - ≥ 5 meiosis; PP1 - ≥ 3 meiosis; and PP3 - concordant in silico tool.

*LQTS* long QT syndrome, *HCM* hypertrophic cardiomyopathy, *CPVT* catecholaminergic polymorphic ventricular tachycardia, *SUD* sudden unexplained death, *MES* MaxEntScan score difference, *ADA* adaptive boosting, *RF* random forest, *P* pathogenic, *VUS* variant of uncertain significance, *LP* likely pathogenic, *PTC* premature termination codon.

### Classification of sequence variants

We classified the pathogenicity of all 88 in silico predicted, splice-disrupting variants; 43 were classified as pathogenic or likely pathogenic and 45 as VUS (Fig. 4, Supplementary Table 4). Most variants in the splice site regions were pathogenic or likely pathogenic, whereas most variants outside these regions were VUS. The results of our RNA-based functional studies, and previously published functional studies, were available for 29 out of 88 variants (33%). They confirmed that 19 variants caused a frameshift leading to a premature termination codon, six caused an in-frame insertion or deletion in the mRNA, one disrupted splicing with unreported consequences, one did not alter splicing, and one resulted in impaired protein function. One variant showed inconsistent results across multiple studies. These mRNA studies supported the reclassification of 11 VUS as likely pathogenic, and 3 likely pathogenic variants were upgraded to pathogenic (Supplementary Table 4). The reclassification of 11 VUS as likely pathogenic allowed these variants to be used for cascade genetic testing. Of these 11 VUS, six variants co-segregated with a concordant phenotype in 10 family members, one heterozygous family member with an unknown clinical status, and one family member who was genotype positive; phenotype negative.

## Discussion

We assessed the burden of splice-disrupting variants in people with inherited heart disease and SUD. We found that 10% of 1242 individuals had an in silico*-*predicted splice-disrupting variant in a phenotypically concordant disease gene. Most of these people had no alternative genetic explanation for their disease. There was an enrichment of splice-disrupting variants in six genes compared to controls, highlighting the importance of this class of variants. Of all splice variants found, half were clinically actionable, and the rest are prime candidates for functional studies to provide additional evidence for-or-against their clinical relevance. We determined that transcripts of 21 definitive inherited heart disease genes can be amplified using blood RNA despite very low expression in blood. Functional analysis of six splice-disrupting variants confirmed that they caused frameshifts or in-frame alterations to the protein-coding sequence. Our findings highlight the significant contribution that splice-disrupting variants make to inherited heart diseases and the value of functional studies in achieving a genetic diagnosis.

Understanding how variants affect the protein sequence is essential for interpreting pathogenicity. Splice-disrupting variants in genes where a loss-of-function is the primary disease mechanism typically caused frameshifts and were enriched in *MYBPC3* in HCM, *TTN* and *FLNC* in DCM, *PKP2* in ACM and *KCNQ1* in LQTS. Loss-of-function variants in these genes would generally classify as likely pathogenic or pathogenic. In contrast, splicing defects in genes where deleterious missense variants prevail tended to cause in-frame deletions and insertions, such as the c.848+1 G > A variant in *RYR2* causing an in-frame exon skipping. The clinical relevance of these in-frame protein length changes is often uncertain. Enrichment of predicted splice-disrupting variants in *MYH7* in people with HCM was unexpected since *MYH7* loss-of-function variants are not usually pathogenic in HCM. There is a reported enrichment of *MYH7* truncating variants in people with left ventricular noncompaction, primarily driven by a c.732+1 G > A splice donor variant^13^. The enrichment in our study was largely driven by a c.2681 A > G variant, which could also be annotated as a Glu849Gly missense change. *MYH7* transcripts could not be amplified from blood RNA and whether this variant alters splicing is yet to be determined; however, the missense change is currently the most likely cause of HCM. While variants at the essential splice dinucleotides are expected to disrupt canonical splicing, variants outside of these dinucleotides have a more uncertain effect, which is challenging for variant interpretation. This variable effect on splicing may explain why the c.341 C > T *TNNT2* variant has shown different outcomes with functional studies using in vitro assays and primary tissue, or this may be due to differences between the assays. Variants that cause multiple outcomes on protein sequence might partly explain phenotypic variability between people harbouring them. A further consideration with variant interpretation is that missense, nonsense, and synonymous variants are often overlooked as causing a splice defect, but they have drastically different effects on the protein sequence. For example, we have previously demonstrated that an assumed benign synonymous *MYBPC3* c.2274 C > T, Gly758Gly variant in a family with HCM creates a new splice site causing truncation of exon 23 in the mRNA^6^.

Deep intronic splice-gain variants are an increasingly recognised cause of HCM, with an *MYBPC3* c.1224-52 G > A variant, alone, responsible for 1% of HCM^6,14,15^. We found three deep intronic splice-gain variants in *MYBPC3* intron 13 in five unrelated people, of whom three had the c.1224-52 G > A variant. Altogether, we found 25 deep intronic in silico-predicted splice variants, 12 of which were found within 100 nucleotides of flanking intron sequence with exome sequencing. The contribution of deep intronic splice variants in our study is likely underestimated, as only 81 people had all intronic regions sequenced with genome sequencing.

We used stringent in silico parameters to predict variants causing a splice defect. RF and ADA tools are limited to evaluating variants at canonical splice sites, whereas MaxEntScan (MES)^16^, and SpliceAI^17^ can be applied genome-wide. For variants at canonical splice sites, all four tools predicted a splicing defect for 25 variants, three tools predicted 11 variants, two tools predicted eight variants, and one tool predicted one variant (Supplementary Table 6). We, or others, have confirmed that variants in each category disrupt splicing. Only MES and SpliceAI tools could evaluate variants outside of canonical splice sites. Thirty variants were predicted to disrupt splicing by MES alone, 8 variants were predicted to disrupt splicing by both tools, and 2 variants were predicted to disrupt splicing by SpliceAI alone. Variants within each category have been confirmed by us, or others, using functional studies. The variability in splicing predictions is likely based on the underlying design and algorithms that each tool uses. Most of these tools are designed to predict whether variants alter splicing at canonical GT/AG splice sites and perform less well with the rare minor introns that begin and end with different dinucleotides, such as AT/AC. We found an *SCN5A* c.4434+5 G > A variant in one such AT/AC minor intron that was predicted to disrupt splicing by SpliceAI, whereas the other tools did not recognise the wild-type splice sequence. Given the prediction variability, multiple in silico tools should be used to identify putative splicing variants.

Our findings have important clinical implications for people and families with inherited heart diseases. In almost all inherited heart diseases, genetic testing currently fails to identify a cause in at least 50% of cases. The recognition of splice-disrupting variants in the current study begins to explain some of these gene-elusive people and families, with very important clinical implications including a more precise diagnosis, guiding therapies, screening at-risk family members, and in providing options for reproductive decisions.

Limitations of our study include that we did not use tools to evaluate variants that disrupt splice branchpoints, enhancers and silencer sequences or in the 5’ and 3’ untranslated regions. Our study focused on definitively disease-associated genes and did not include genes with more recently reported disease associations, such as *ALPK3*^18^, and *FHOD3*^19,20^. We were underpowered to confirm a significant enrichment of splice variants in *SCN5A* in people with BrS. Only 81 people had genome sequencing, so the contribution of deep intronic splice-disrupting variants may be underestimated.

In conclusion, splice-disrupting variants are a significant cause of inherited heart diseases and SUD. Missense, nonsense and synonymous should be evaluated for possible effects on splicing as this can impact the interpretation of pathogenicity. Blood RNA is an accessible surrogate for primary heart tissue for RNA testing of many inherited heart disease genes, which increases the diagnostic yield of genetic testing. Confirmation of a deleterious splicing outcome in families with inherited heart disease and SUD has immediate clinical benefits, including guiding treatment options and facilitating risk stratification in family members.

## Methods

### Study cohort

People with inherited heart disease or sudden unexplained death (SUD) were recruited from the Genetic Heart Disease and Hypertrophic Cardiomyopathy Clinics, Royal Prince Alfred Hospital, Sydney, Australia and the Royal Children’s Hospital, Melbourne, Australia. We included people diagnosed with ACM (MIM 607450), DCM (MIM 604145), HCM (MIM 613426), BrS (MIM 601144), CPVT (MIM 604772), LQTS (MIM 613688), or SUD^3^. We excluded people with restrictive cardiomyopathy and isolated left ventricular non-compaction as ClinGen curation of disease-gene associations had not been performed. All participants were enrolled in protocol X20-0157/ETH00776, approved by the Sydney Local Health District Ethics Review Committee, Australia, or protocol #32092, approved by The Royal Children’s Hospital Melbourne Research Ethics Committee. Written informed consent was provided by participants, parents for enrolled children or by next of kin for enrolled deceased participants.

### In silico prioritisation of splice-altering variants

All participants had undergone prior genetic testing with cardiac gene panel (36%), exome (57%), or genome sequencing (7%), as previously described^6,21^. Variants were retrieved in phenotypically concordant and definitively disease-associated genes, as established by the ClinGen Gene Expert panels^22–27^, except for SUD, in which we retrieved variants in all 32 definitively disease-associated genes (Supplementary Table 7). Candidate splice-disrupting variants were required to have an allele frequency <0.0001 in the Genome Aggregation Database (gnomAD)^28,29^, equating to an allele count ≤15 in gnomAD v2.1.1 and v3.1.2. In silico splice prediction tools MES^16^, ADA, RF^12^, and SpliceAI^17^ were used to identify putative splicing variants as they are readily available with Ensembl’s Variant Predictor and have been shown to offer sensitivity and specificity for splice site variants in cardiac genes^30^. Variants were selected if they were located in a canonical splice site and reduced the MES score by >4, or created a new splice site with an MES score >4 and showed an increase of >4 from wild-type sequence^31^, or if they scored >0.6 with ADA or RF^12^, or >0.5 with SpliceAI^17^, as recommended by the developers of the tools.

### Rare splice variant burden testing

Rare splice variant burden testing between case cohorts and gnomAD exomes, v2.1.1 was performed as per Mazzarotto et al. (2021)^13^. Rare variants were defined as having an African, East Asian, Latino, non-Finnish European and South Asian sub-population allele frequency and overall allele frequency in gnomAD exomes <0.0001. Variants must also be in protein-coding regions or within the first and last 75 bp of intron sequence to account for exome sequencing target regions. We excluded the *TNNT2* NM_001276345.2: c.601-1 G > A variant, found in 5 participants with Oceanian ancestry, as this is a rare Oceanian polymorphism. The case denominator was the number of individuals within each disease group, and for gnomAD, the denominator was the average number of people sequenced for each gene to account for variable coverage in exome-sequenced samples. Statistical significance for the enrichment of variants in cases was assessed with a one-sided Fisher’s exact test, with Bonferroni correction applied for testing the number of definitively disease-associated genes for each disease. The case excess was defined as the difference in rare variant frequencies between cases and gnomAD. Power calculations were performed using the ES.h() and pwr.2p2n.test() functions of the R package pwr.

### RNA extraction

RNA was extracted from 2.5 mL of peripheral blood collected in PAXGene^®^ Blood RNA Tubes (PreAnalytiX, Hombrechtikon, Switzerland) using the PAXGene^®^ blood RNA kit (PreAnalytiX) according to manufacturer’s instructions, except for a final elution in 40 µL of elution buffer. RNA was extracted from induced pluripotent stem cell-derived cardiomyocytes from two unrelated people, as previously described^32^. RNA was extracted from 20 µg of snap-frozen myectomy tissue homogenised in 1 mL of TRIzol^®^ reagent using the phenol-chloroform method according to the manufacturer’s instructions, except for a final resuspension in 30 µL of diethyl pyrocarbonate (DEPC) treated water. RNA samples were quantified using the Nanodrop ND1000 (Thermo Fisher Scientific, Massachusetts, United States) and assessed with 2% agarose gel electrophoresis stained with 1% Gel Red using the GelDoc Go System (BioRad, California, United States).

### RT-PCR amplification of definitively disease-associated cardiac genes

Primers were designed to amplify at least three consecutive exons of MANE (matched annotation between NCBI and EBI) transcripts, with an RT-PCR product size between 340 to 500 bp, except for *BAG3*, *KCNH2* and *TTN*, which amplified larger products due to large exon sizes. One microgram of RNA was reverse transcribed using 200 U Superscript™ III (Invitrogen, Massachusetts, United States), 4 µl Superscript III™ 5X first-strand buffer, 5 mM DTT, 40 U RNaseOUT™ (Thermo-Fisher), 0.25 mM dNTP (Roche, Basel, Switzerland) and 0.01 mM random hexamer primer (Thermo-Fisher) in a final volume of 20 µL. Complementary DNA products were diluted 1:2 in DEPC-treated water and amplified as previously described^5^, using an annealing temperature of 60 °C unless otherwise specified (Supplementary Table 5). PCR products were resolved on an agarose gel and all gel images were derived from the same experiment. PCR products were purified using 5 U Antarctic Phosphatase (New England BioLabs, Massachusetts, United States) and 20 U Exonuclease I (New England BioLabs), and Sanger sequenced at the Macrogen Sequencing Facility (Seoul, Republic of Korea). Chromatograms were analysed using Sequencher™ v.5.4.6 (Gene Codes Corporation, Michigan, United States).

### Functional studies of splice-altering variants

Functional studies of putative splicing variants in genes that successfully amplified in blood RNA was performed on 500 ng RNA extracted from the participant’s fresh blood using the PAXGene^®^ blood RNA kit. Functional studies were performed as previously described^5^. PCR primer sequences are shown in Supplementary Table 8.

### Classification of variants using the ACMG/AMP framework

All putative splice variants were classified for pathogenicity using the ACMG/AMP classification framework^33^. PVS1 was assigned to variants causing a frameshift in genes where loss-of-function is an established disease mechanism. We also applied PVS1 to loss-of-function variants in genes with a ClinGen gene dosage haploinsufficiency score of 3, which represents the highest rating given to genes with sufficient evidence supporting a dosage sensitivity (https://search.clinicalgenome.org/kb/gene-dosage). PVS1_strong was given to genes with a haploinsufficiency score of 2 and to titin (*TTN*), representing genes with emerging evidence of dosage sensitivity. PVS1_moderate was applied to genes with a haploinsufficiency score of 1, representing little evidence of dosage sensitivity. PVS1_supporting was applied to genes that have not yet been curated for dosage sensitivity but have a ratio of <0.35 for the observed to expected (o/e) number of loss-of-function variants in that gene, as provided by gnomAD.

### Reporting summary

Further information on research design is available in the Nature Research Reporting Summary linked to this article.

### Supplementary information

Supplementary Figures

Supplementary Data

REPORTING SUMMARY

## Data Availability

Data used and/or analysed during the current study is provided in the Supplementary information, original gel images and Sanger sequencing files are available from the corresponding author. Gene panel testing was performed by a clinically accredited testing facility, Victorian Clinical Genetics Services (Melbourne, Australia), and therefore unavailable. Whole exome and genome sequencing data are subject to conditions of Ethics agreement X20-0157/ETH00776 under which the data was generated, and therefore unavailable unless a data sharing agreement has been obtained from the Sydney Local Health District Ethics Review Committee, Australia. Control dataset was obtained from gnomAD (https://gnomad.broadinstitute.org/).

## References

[1] Al-Khatib SM (2018). 2017 AHA/ACC/HRS Guideline for Management of Patients With Ventricular Arrhythmias and the Prevention of Sudden Cardiac Death: Executive Summary. Circulation.

[2] McKenna WJ, Maron BJ, Thiene G (2017). Classification, Epidemiology, and Global Burden of Cardiomyopathies. Circ. Res..

[3] Wilde AAM (2022). European Heart Rhythm Association (EHRA)/Heart Rhythm Society (HRS)/Asia Pacific Heart Rhythm Society (APHRS)/Latin American Heart Rhythm Society (LAHRS) Expert Consensus Statement on the state of genetic testing for cardiac diseases. EP Eur..

[4] Patel PN (2021). Contribution of Noncanonical Splice Variants to TTN Truncating Variant Cardiomyopathy. Circ. Genom. Precis. Med..

[5] Singer ES, Ingles J, Semsarian C, Bagnall RD (2019). Key Value of RNA Analysis of MYBPC3 Splice-Site Variants in Hypertrophic Cardiomyopathy. Circ. Genom. Precis. Med..

[6] Bagnall RD (2018). Whole Genome Sequencing Improves Outcomes of Genetic Testing in Patients With Hypertrophic Cardiomyopathy. J. Am. Coll. Cardiol..

[7] Shapiro MB, Senapathy P (1987). RNA splice junctions of different classes of eukaryotes: sequence statistics and functional implications in gene expression. Nucleic Acids Res..

[8] Zhang MQ (1998). Statistical Features of Human Exons and Their Flanking Regions. Hum. Mol. Genet..

[9] Riepe TV (2021). Benchmarking deep learning splice prediction tools using functional splice assays. Hum. Mutat..

[10] Richards S (2015). Standards and guidelines for the interpretation of sequence variants: a joint consensus recommendation of the American College of Medical Genetics and Genomics and the Association for Molecular Pathology. Genet. Med..

[11] Bournazos AM (2022). Standardized practices for RNA diagnostics using clinically accessible specimens reclassifies 75% of putative splicing variants. Genet. Med..

[12] Jian X, Boerwinkle E, Liu X (2014). In silico prediction of splice-altering single nucleotide variants in the human genome. Nucleic Acids Res..

[13] Mazzarotto F (2021). Systematic large-scale assessment of the genetic architecture of left ventricular noncompaction reveals diverse etiologies. Genet. Med..

[14] Holliday M (2021). Transcriptome Sequencing of Patients With Hypertrophic Cardiomyopathy Reveals Novel Splice-Altering Variants in *MYBPC3*. Circ. Genom. Precis. Med..

[15] Harper AR (2020). Reevaluation of the South Asian MYBPC3Δ25bp Intronic Deletion in Hypertrophic Cardiomyopathy. Circ. Genom. Precis. Med..

[16] Yeo G, Burge CB (2004). Maximum Entropy Modeling of Short Sequence Motifs with Applications to RNA Splicing Signals. J. Comput. Biol..

[17] Jaganathan K (2019). Predicting Splicing from Primary Sequence with Deep Learning. Cell.

[18] Lopes LR (2021). Alpha-protein kinase 3 (ALPK3) truncating variants are a cause of autosomal dominant hypertrophic cardiomyopathy. Eur. Heart J..

[19] Ochoa JP (2018). Formin Homology 2 Domain Containing 3 (FHOD3) Is a Genetic Basis for Hypertrophic Cardiomyopathy. J. Am. Coll. Cardiol..

[20] Semsarian C, Ingles J, Bagnall RD (2019). Revisiting Genome Sequencing Data in Light of Novel Disease Gene Associations. J. Am. Coll. Cardiol..

[21] Bagnall RD (2016). Exome-based analysis of cardiac arrhythmia, respiratory control, and epilepsy genes in sudden unexpected death in epilepsy. Ann. Neurol..

[22] Adler A (2020). An International, Multicentered, Evidence-Based Reappraisal of Genes Reported to Cause Congenital Long QT Syndrome. Circulation.

[23] Hosseini SM (2018). Reappraisal of Reported Genes for Sudden Arrhythmic Death. Circulation.

[24] Ingles J (2019). Evaluating the Clinical Validity of Hypertrophic Cardiomyopathy Genes. Circ. Genom. Precis. Med..

[25] James CA (2021). International Evidence Based Reappraisal of Genes Associated With Arrhythmogenic Right Ventricular Cardiomyopathy Using the Clinical Genome Resource Framework. Circ. Genom. Precis. Med..

[26] Jordan E (2021). Evidence-Based Assessment of Genes in Dilated Cardiomyopathy. Circulation.

[27] Walsh R (2022). Evaluation of gene validity for CPVT and short QT syndrome in sudden arrhythmic death. Eur. Heart J..

[28] Whiffin N (2017). Using high-resolution variant frequencies to empower clinical genome interpretation. Genet. Med..

[29] Karczewski KJ (2020). The mutational constraint spectrum quantified from variation in 141,456 humans. Nature.

[30] Rayani, K. et al. Identification and in-silico characterization of splice-site variants from a large cardiogenetic national registry. *Journal*; 10.1038/s41431-022-01193-9 (2022). 10.1038/s41431-022-01193-9 PMC10172209 36138163

[31] Ito K (2017). Identification of pathogenic gene mutations in LMNA and MYBPC3 that alter RNA splicing. Proc. Natl Acad. Sci. USA..

[32] Holliday M, Ross SB, Lim S, Semsarian C (2018). Generation of an induced pluripotent stem cell line from a hypertrophic cardiomyopathy patient with a pathogenic myosin binding protein C (MYBPC3) p.Arg502Trp mutation. Stem Cell Res..

[33] Kelly MA (2018). Adaptation and validation of the ACMG/AMP variant classification framework for MYH7-associated inherited cardiomyopathies: recommendations by ClinGen’s Inherited Cardiomyopathy Expert Panel. Genet. Med..

