# Supplementary material for: The burden of splice-disrupting variants in inherited heart disease and unexplained sudden cardiac death
Source: NPJ Genom Med. 2023 Oct 11;8:29. doi: 10.1038/s41525-023-00373-w (PMC10567745; doi:10.1038/s41525-023-00373-w)
Supplement: Supplementary file 1 — Supplementary Figures [file 41525_2023_373_MOESM1_ESM.pdf]

The burden of splice-disrupting variants in inherited heart disease and unexplained sudden cardiac death

Emma S. Singer,[1,2] Joshua Crowe,[1,2] Mira Holliday,[1,2] Julia C. Isbister,[1-3] Sean Lal,[2] Natalie Nowak,[1,3] Laura Yeates,[1-6] Charlotte Burns,[1,3] Sulekha Rajagopalan,[7] Ivan Macciocca,[8-10] Ingrid King,[8] Julie Wacker,[11] Jodie Ingles,[2-6] Robert G Weintraub, [8,10,11] Christopher Semsarian,[1-3] Richard D. Bagnall.[1,2]

Supplementary Fig. 1. Conserved sequences at exon-intron junctions and possible splicing outcomes. Page 2.

Supplementary Fig. 2. Evaluation of mRNA sources for functional studies of cardiac disease genes. Pages 3 to 7.

Supplementary Fig. 3. Functional studies of *in silico*-predicted splice-disrupting variants. Pages 8 to 9.

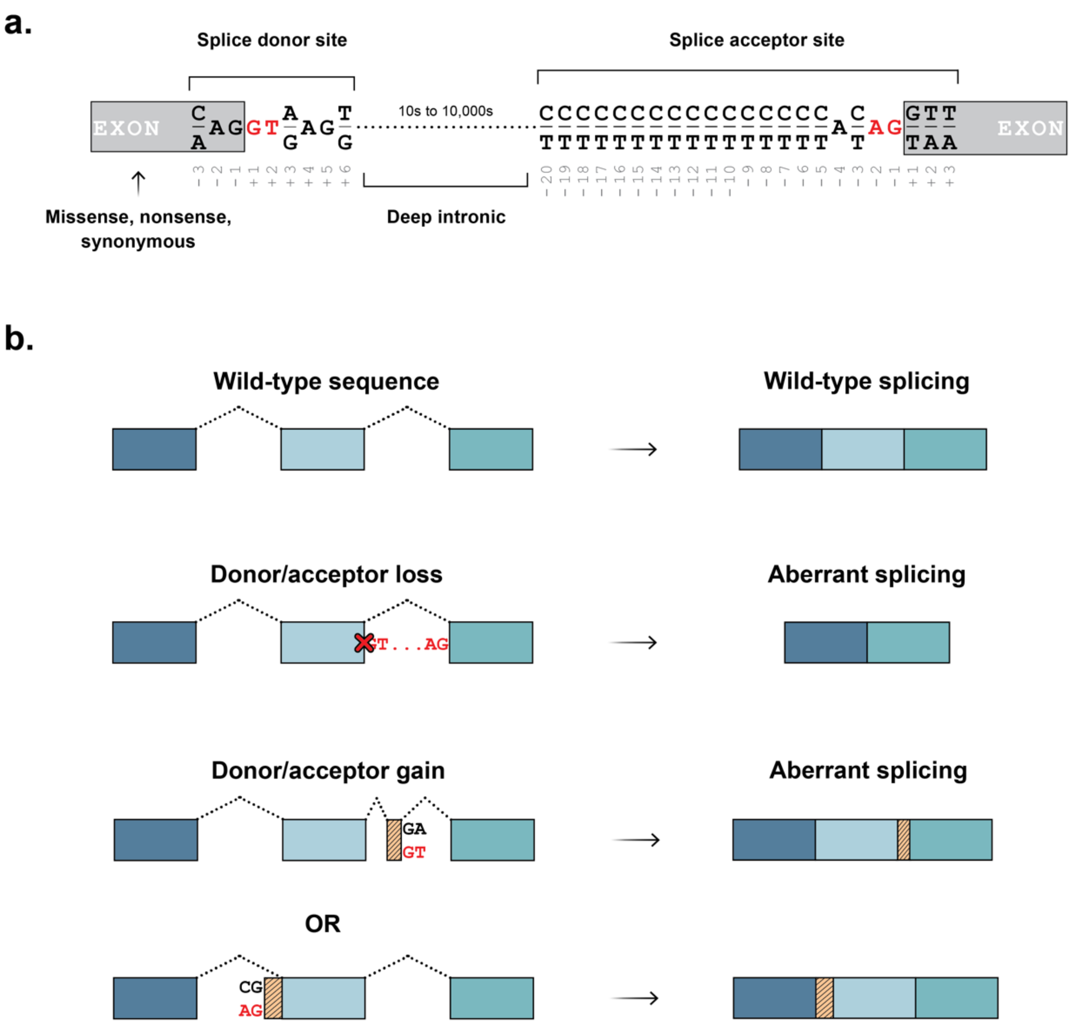

Supplementary Fig. 1. Conserved sequences at exon-intron junctions and possible splicing outcomes. (a) Location and numbering of consensus sequences at essential splice-site junctions. The essential dinucleotides (GT/AG) marking the donor and acceptor sites are shown in red. Exons are shown as grey boxes. **(b)** Aberrant splice products demonstrate the possible functional consequence of each type of splice-disrupting variant. Exons are shown as coloured boxes. Dotted lines indicate splicing patterns. Inclusion of a pseudo-exon is shown as orange hatched boxes. The essential dinucleotides (GT/AG) are shown in red.

Supplementary Fig. 2.

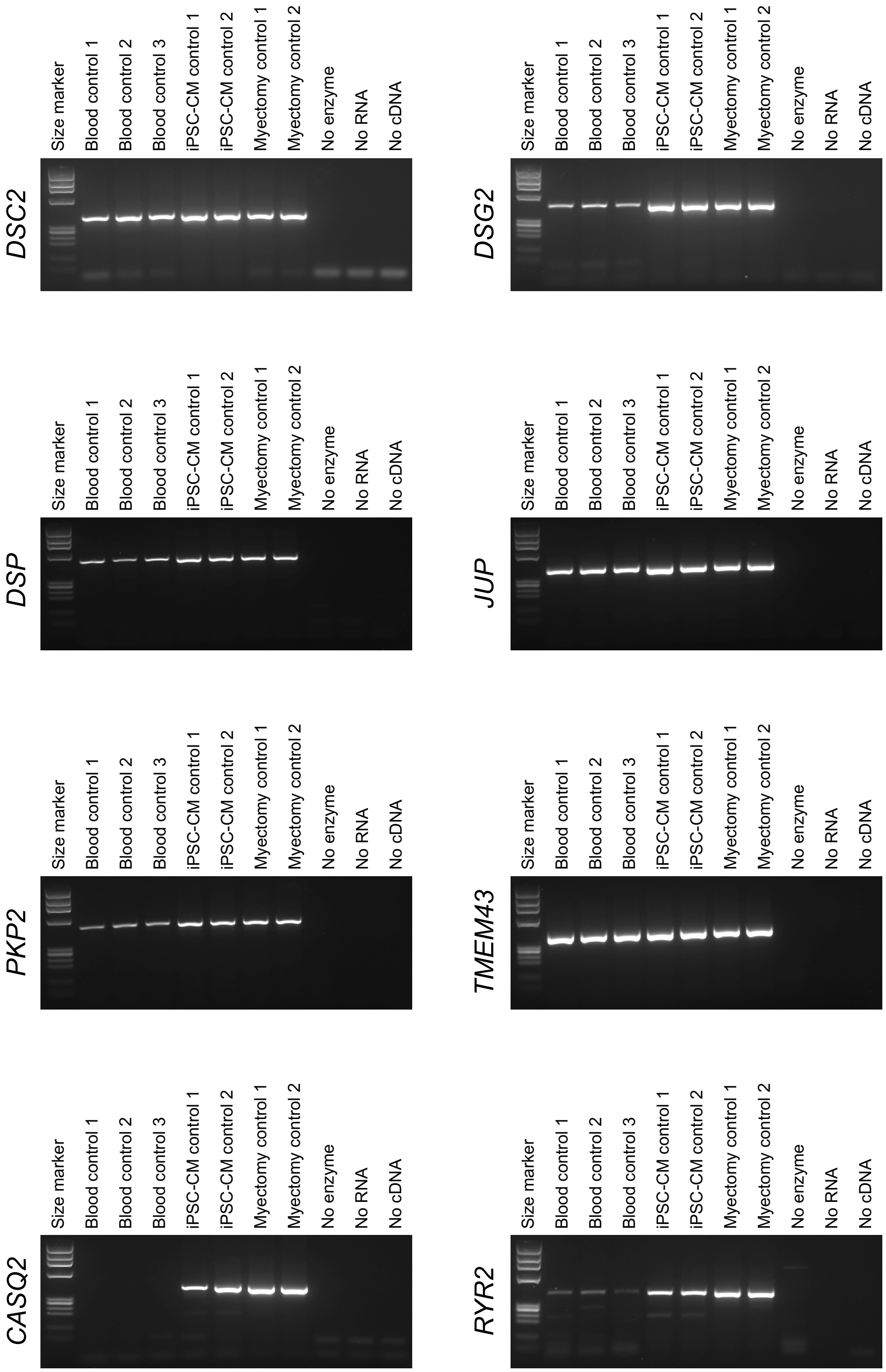

Supplementary Fig. 2 continued.

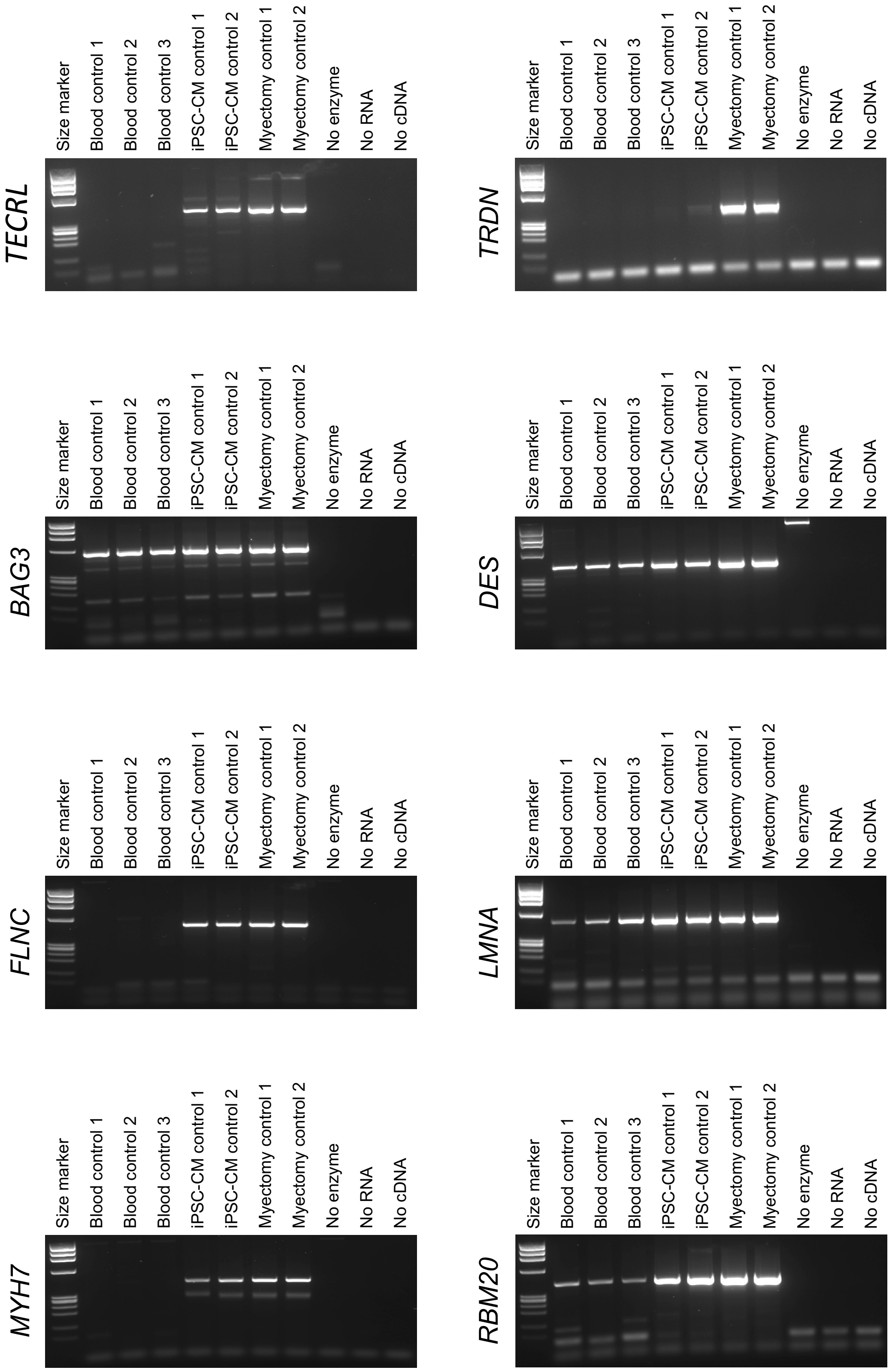

Supplementary Fig. 2 continued.

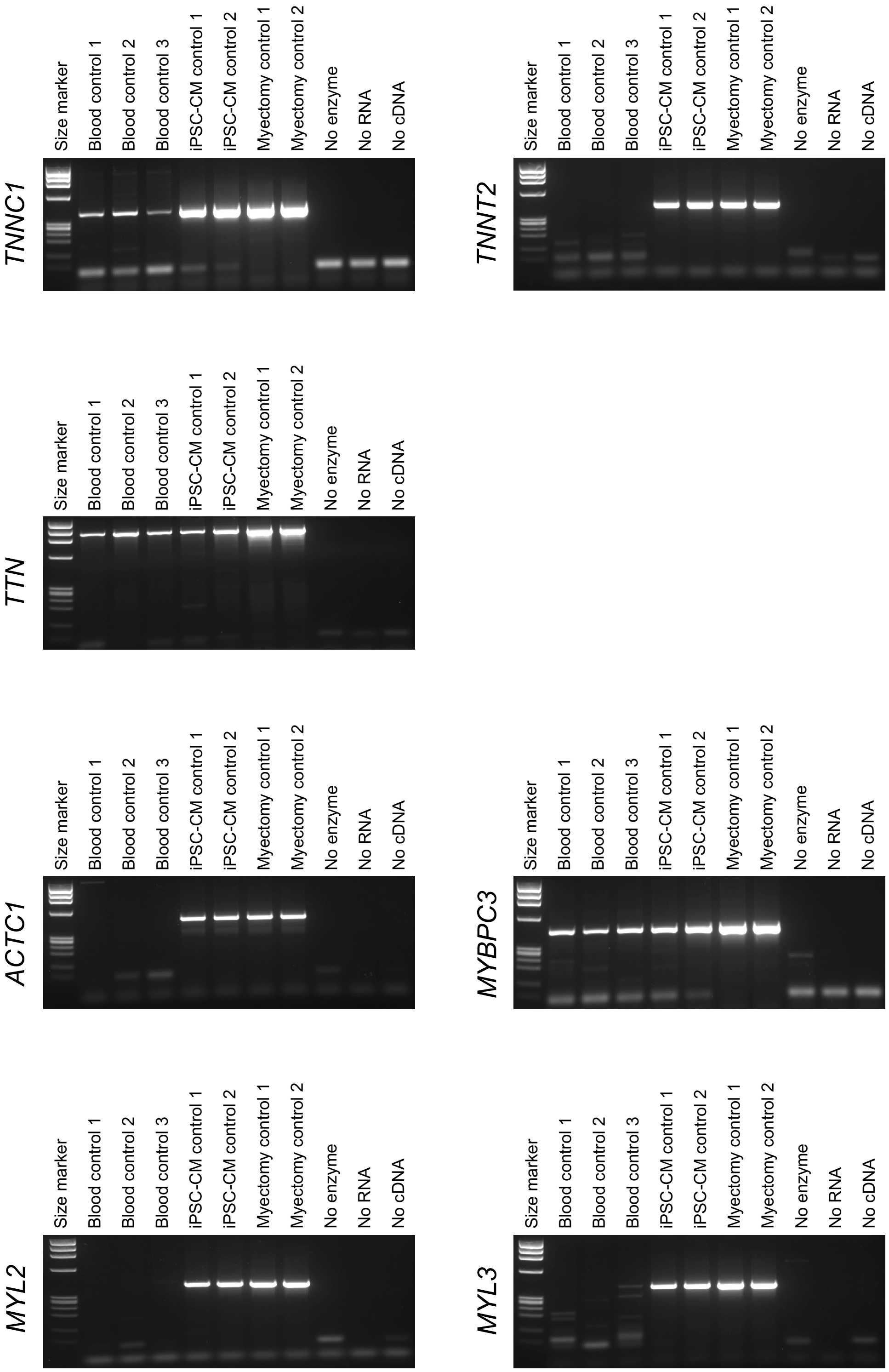

Supplementary Fig. 2 continued.

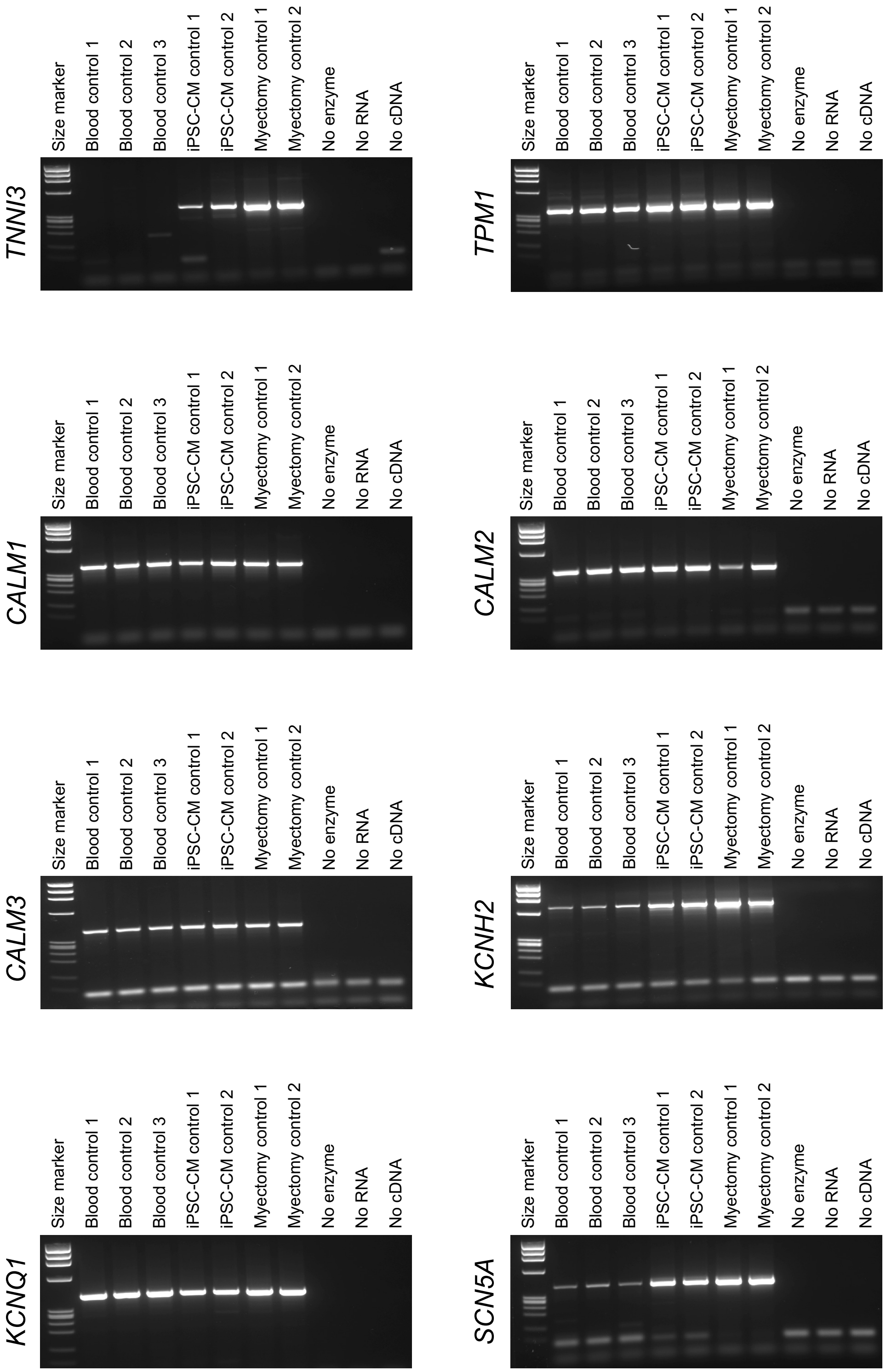

Supplementary Fig. 2. Evaluating RNA sources for functional analysis of cardiac disease genes. Thirty-one definitively associated disease genes were RT-PCR amplified using RNA extracted from fresh blood, induced pluripotent stem cell cardiomyocytes and myectomy tissue. The Clinical Genome Resource (https://clinicalgenome.org/) classified the genes as a definitive cause of arrhythmogenic cardiomyopathy - *DSC2, DSG2, DSP, JUP, PKP2, TMEM43;* dilated cardiomyopathy - *BAG3, DES, FLNC, LMNA, MYH7, RBM20, TNNC1, TNNT2, TTN;* hypertrophic cardiomyopathy - *ACTC1, MYBPC3, MYH7, MYL2, MYL3, TNNI3, TNNT2, TPM1;* Brugada syndrome – *SCN5A*; catecholaminergic polymorphic ventricular tachycardia – *CASQ2, RYR2, TECRL, TRDN*; and long QT syndrome – *CALM1, CALM2, CALM3, KCNH2, KCNQ1, SCN5A.* No enzyme – no reverse transcriptase negative control; No RNA – no RNA negative control; No cDNA – no cDNA negative control. Each gel image is derived from the same experiment.

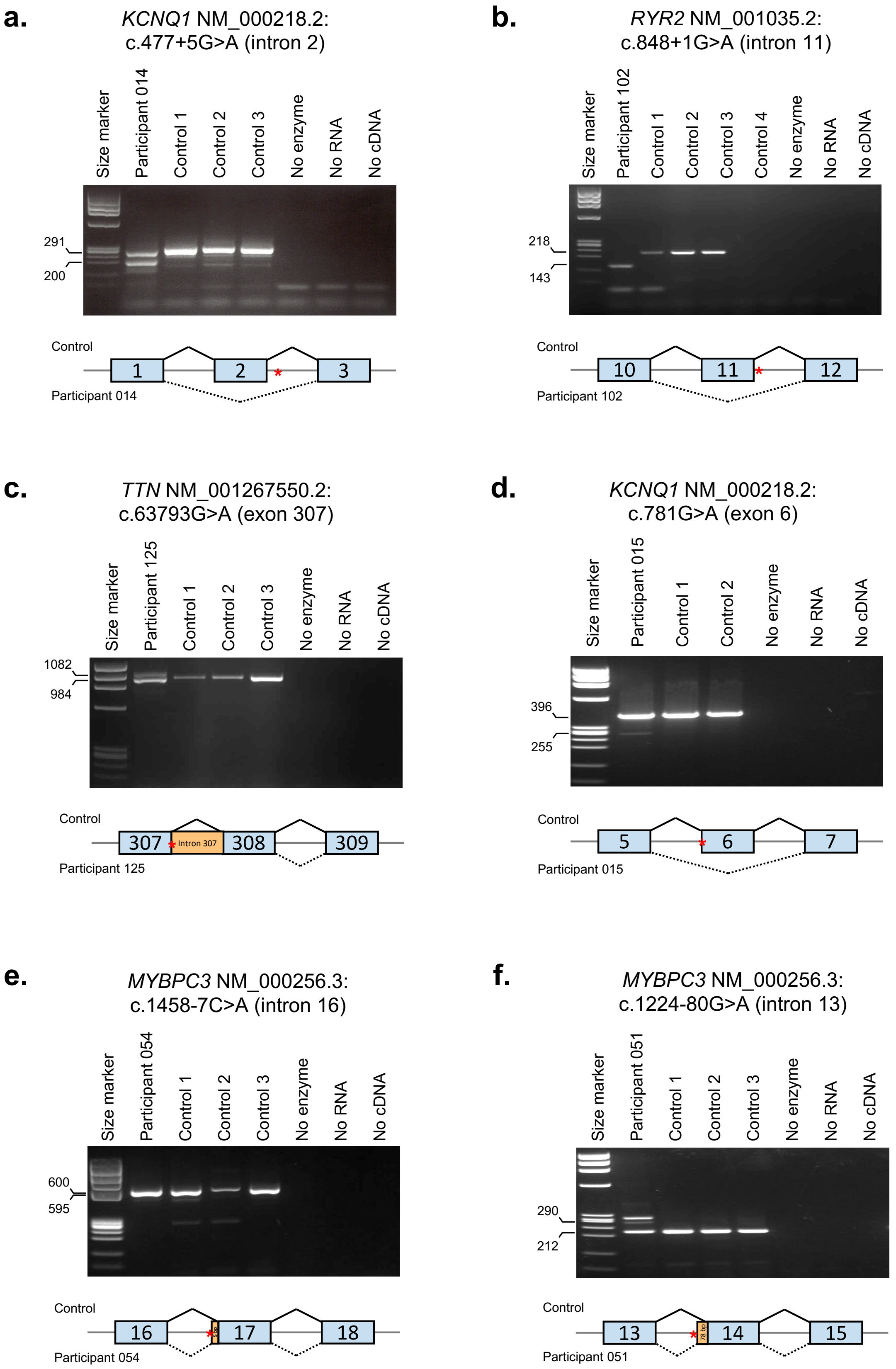
a.
KCNQ1 NM_000218.2:
c.477+5G>A (intron 2)
Size marker
Participant 014
Control 1
Control 2
Control 3
No enzyme
No RNA
No cDNA
291
200
Control
1
2
3
Participant 014
b.
RYR2 NM_001035.2:
c.848+1G>A (intron 11)
Size marker
Participant 102
Control 1
Control 2
Control 3
Control 4
No enzyme
No RNA
No cDNA
218
143
Control
10
11
12
Participant 102
c.
TTN NM_001267550.2:
c.63793G>A (exon 307)
Size marker
Participant 125
Control 1
Control 2
Control 3
No enzyme
No RNA
No cDNA
1082
984
Control
307
Intron 307
308
309
Participant 125
d.
KCNQ1 NM_000218.2:
c.781G>A (exon 6)
Size marker
Participant 015
Control 1
Control 2
No enzyme
No RNA
No cDNA
396
255
Control
5
6
7
Participant 015
e.
MYBPC3 NM_000256.3:
c.1458-7C>A (intron 16)
Size marker
Participant 054
Control 1
Control 2
Control 3
No enzyme
No RNA
No cDNA
600
595
Control
16
17
18
Participant 054
f.
MYBPC3 NM_000256.3:
c.1224-80G>A (intron 13)
Size marker
Participant 051
Control 1
Control 2
Control 3
No enzyme
No RNA
No cDNA
290
212
Control
13
78 bp
14
15
Participant 051

Supplementary Fig. 3. Functional studies of *in silico*-predicted splice-disrupting variants. Panels show gel electrophoresis of RT-PCR outcomes for variants located in the splice-donor site **(a, b and c)**, splice-acceptor site **(d and e)**, and a deep intronic variant **(f)**. RNA splicing outcomes were assessed in participants and 3 control individuals who do not have the assessed variant using RNA extracted from collected fresh venous blood. Canonical exons are shown as blue boxes, introns as black horizontal lines, and pseudo-exons as orange boxes. Normal splicing is depicted by solid black lines and disease-associated splicing by dotted lines. Red asterisks indicate the approximate location of the assessed variant. No enzyme – no reverse transcriptase negative control; No RNA – no RNA negative control; No cDNA – no cDNA negative control. Each gel image is derived from the same experiment.
