# Supplementary material for: The burden of splice-disrupting variants in inherited heart disease and unexplained sudden cardiac death
Source: NPJ Genom Med. 2023 Oct 11;8:29. doi: 10.1038/s41525-023-00373-w (PMC10567745; doi:10.1038/s41525-023-00373-w)
Supplement: Supplementary file 3 — REPORTING SUMMARY [file 41525_2023_373_MOESM3_ESM.pdf]

nature portfolio

Corresponding author(s): Richard Bagnall
Last updated by author(s): Sep 13, 2023

Reporting Summary

Nature Portfolio wishes to improve the reproducibility of the work that we publish. This form provides structure for consistency and transparency in reporting. For further information on Nature Portfolio policies, see our Editorial Policies and the Editorial Policy Checklist.

Statistics

For all statistical analyses, confirm that the following items are present in the figure legend, table legend, main text, or Methods section.

n/a	Confirmed	
☐	☒	The exact sample size (*n*) for each experimental group/condition, given as a discrete number and unit of measurement
☒	☐	A statement on whether measurements were taken from distinct samples or whether the same sample was measured repeatedly
☐	☒	The statistical test(s) used AND whether they are one- or two-sided<br>*Only common tests should be described solely by name; describe more complex techniques in the Methods section.*
☒	☐	A description of all covariates tested
☐	☒	A description of any assumptions or corrections, such as tests of normality and adjustment for multiple comparisons
☐	☒	A full description of the statistical parameters including central tendency (e.g. means) or other basic estimates (e.g. regression coefficient) AND variation (e.g. standard deviation) or associated estimates of uncertainty (e.g. confidence intervals)
☒	☐	For null hypothesis testing, the test statistic (e.g. *F*, *t*, *r*) with confidence intervals, effect sizes, degrees of freedom and *P* value noted<br>*Give P values as exact values whenever suitable.*
☒	☐	For Bayesian analysis, information on the choice of priors and Markov chain Monte Carlo settings
☒	☐	For hierarchical and complex designs, identification of the appropriate level for tests and full reporting of outcomes
☐	☒	Estimates of effect sizes (e.g. Cohen's *d*, Pearson's *r*), indicating how they were calculated

Our web collection on statistics for biologists contains articles on many of the points above.

Software and code

Policy information about availability of computer code

Data collection	Exome and genome sequencing data were aligned to the GRCh38 human reference sequence using BWA-MEM v0.7.17, converted to binary format using SAMtools5, and sorted with duplicate read removal using NovoSort (Novocraft Technologies, Selangor, Malaysia). Aligned reads underwent base recalibration, haplotype calling, importing into a genomic database, genotyping and variant quality score recalibration following the GATK v4.1.9.0 best practice guidelines.
Data analysis	Splicing variant analysis was performed using MaxEntScan, SpliceAI and dbscSNV, which are available in web-based access through the Variant Effect Predictor at https://asia.ensembl.org/Tools/VEP. Power calculations were performed using the ES.h() and pwr.2p2n.test() functions of the R package pwr, V1.3-0.

For manuscripts utilizing custom algorithms or software that are central to the research but not yet described in published literature, software must be made available to editors and reviewers. We strongly encourage code deposition in a community repository (e.g. GitHub). See the Nature Portfolio guidelines for submitting code & software for further information.

Data

Policy information about availability of data

All manuscripts must include a data availability statement. This statement should provide the following information, where applicable:

- Accession codes, unique identifiers, or web links for publicly available datasets
- A description of any restrictions on data availability
- For clinical datasets or third party data, please ensure that the statement adheres to our policy

Data used and/or analysed during the current study is provided in the Supplementary information, original gel images and Sanger sequencing files are available from the corresponding author. Gene panel testing was performed by a clinically accredited testing facility, Victorian Clinical Genetics Services (Melbourne, Australia), and therefore unavailable. Whole exome and genome sequencing data are subject to conditions of Ethics agreement X20-0157/ETH00776 under which the data was generated, and therefore unavailable unless a data sharing agreement has been obtained from the Sydney Local Health District Ethics Review Committee, Australia. Control dataset was obtained from gnomAD (https://gnomad.broadinstitute.org/).

Research involving human participants, their data, or biological material

Policy information about studies with human participants or human data. See also policy information about sex, gender (identity/presentation), and sexual orientation and race, ethnicity and racism.

Reporting on sex and gender	The cohort is 66.5% male and 33.5% female based on self-reporting sex. Sex disaggregated analysis is not applicable.
Reporting on race, ethnicity, or other socially relevant groupings	Self-reported ethnicities were available for 122 participants with splice-disrupting variants, with 95 European, 12 Asian, 6 North African, 8 Oceanian, and 1 'Peoples of the Americas'
Population characteristics	No covariate-relevant population characteristics were used.
Recruitment	Patients were recruited from a tertiary referral centre.
Ethics oversight	All participants were enrolled in protocol X20-0157/ETH00776, approved by the Sydney Local Health District Ethics Review Committee, Australia, or protocol #32092, approved by The Royal Children's Hospital Melbourne Research Ethics Committee.

Note that full information on the approval of the study protocol must also be provided in the manuscript.

Field-specific reporting

Please select the one below that is the best fit for your research. If you are not sure, read the appropriate sections before making your selection.

☒ Life sciences ☐ Behavioural & social sciences ☐ Ecological, evolutionary & environmental sciences

For a reference copy of the document with all sections, see nature.com/documents/nr-reporting-summary-flat.pdf

Life sciences study design

All studies must disclose on these points even when the disclosure is negative.

Sample size	All available individuals with genetic testing available were included. Power calculations for burden testing are provided in supplementary tables.
Data exclusions	Individuals without genetic testing results were excluded from our study. We excluded the TNNT2 NM_001276345.2:c.601-1G>A variant from our burden testing analysis, found in 5 participants with Oceanian ancestry, as this is a rare Oceanian polymorphism
Replication	No replication was performed as only one patient cohort was available.
Randomization	Not applicable
Blinding	Blinding was not applicable to this observationbal study

Reporting for specific materials, systems and methods

We require information from authors about some types of materials, experimental systems and methods used in many studies. Here, indicate whether each material, system or method listed is relevant to your study. If you are not sure if a list item applies to your research, read the appropriate section before selecting a response.

Materials & experimental systems

n/a	Involved in the study
☒	☐ Antibodies
☒	☐ Eukaryotic cell lines
☒	☐ Palaeontology and archaeology
☒	☐ Animals and other organisms
☒	☐ Clinical data
☒	☐ Dual use research of concern
☒	☐ Plants

Methods

n/a	Involved in the study
☒	☐ ChIP-seq
☒	☐ Flow cytometry
☒	☐ MRI-based neuroimaging
